# Machining of Lenticular Lens Silicon Molds with a Combination of Laser Ablation and Diamond Cutting

**DOI:** 10.3390/mi10040250

**Published:** 2019-04-16

**Authors:** Jide Han, Lihua Li, Wingbun Lee

**Affiliations:** The State Key Laboratory of Ultraprecision Machining Technology, Department of Industrial and Systems Engineering, The Hong Kong Polytechnic University, Kowloon, Hong Kong, China; jide.han@connect.polyu.hk (J.H.); wb.lee@polyu.edu.hk (W.L.)

**Keywords:** hybrid machining, laser ablation, diamond cutting, lenticular lens, single crystal silicon

## Abstract

Lenticular lenses are widely used in the three-dimensional display industry. Conventional lenticular lens components are made of plastics that have low thermal stability. An alternative is to use glass to replace plastic as the lenticular lens component material. Single crystal silicon is often used as the mold material in the precision glass molding process. It is, however, difficult to fabricate a lenticular lens silicon mold that has a large feature size compared to the critical depth of cut of silicon. In order to solve the problems of machining lenticular lens silicon molds using the conventional diamond cutting method, such as low machining efficiency and severe tool wear, a hybrid machining method that combined laser ablation and diamond cutting was proposed. A feasibility study was performed to investigate the possibility of using this method to fabricate a lenticular lens silicon mold. The influence of the laser parameters and machining parameters on the machining performance was investigated systematically. The experimental results indicated that this hybrid machining method could be a possible method for manufacturing lenticular lens silicon molds or other similar microstructures.

## 1. Introduction

Autostereoscopic display technology is a glass-free, three-dimensional (3D) technology that allows people to acquire a 3D perception of images without wearing specialized headgear or glasses. There are mainly two types of autostereoscopic display techniques: the parallax barrier type and the lenticular lens type [1]. With the parallax barrier type display, 50% of the light that each eye can observe is blocked by the barrier. Therefore, there is a great light efficiency loss in this type of display. For the lenticular lens type, the 3D effect is realized by the refraction function of lenticular lenses, which can deliver different images to the right and left eyes separately. Light is not blocked, thus the throughput of light in the lenticular lens display is much higher than that of the parallax barrier display. Conventionally, the lenticular lens is made of plastic using the injection molding method. However, the reliability of this kind of display is low because the thermal expansion between the lenticular lens and the liquid crystal display (LCD) panel is different, thus causing a location shift between them [2]. A lenticular lens made of glass is therefore needed for high quality large 3D-LCD panels.

Precision glass molding is a promising method in the large-scale industrial production of lenticular lenses. Single crystal silicon is an important mold material used in the precision glass molding industry due to its high thermal stability and available processing methods [3,4,5]. However, silicon is a brittle material that is relatively difficult to cut using a diamond tool because cracks are easily formed on the machined surfaces during the cutting process [6,7]. By controlling the depth of cut to less than a critical value, crack-free machining can be achieved [8,9,10,11,12]. Since the critical depth of cut of single crystal silicon is only a few hundred nanometers [13], it is still a rather time consuming process to fabricate microstructures with feature sizes much larger than the value of the critical depth of cut. A lenticular lens is such a microstructure. The depth of the lenticular lens is typically in the order of tens of micrometers [14]. This means that generating this kind of microstructure on a silicon workpiece surface with the ductile region machining method will have extremely low efficiency. It is not difficult to estimate that even creating one groove will require hundreds of passes with diamond cutting. Therefore, the tool wear problem will be very serious due to the long tool workpiece contact time.

The poor machinability of silicon leads to low machining efficiency. There are several approaches to improve the machinability of silicon, such as increasing the critical depth of cut through modifying the material property of the cutting region with the ion implantation method [15,16], or using a laser to assist machining in the diamond cutting process [17,18]. These methods, however, can only improve the machining performance of silicon to a limited extent.

Nonconventional machining, or hybrid machining methods, are also possible alternatives to the diamond cutting method in generating large feature sized microstructures on brittle materials. Youn et al. [19] demonstrated the possibility of using focused ion beam (FIB) milling to produce microstructures with good surface quality (Ra = 4–30 nm) on glassy carbon though the efficiency is very low. Kim et al. [20] successfully fabricated a lenticular lens fused silica mold, which was then used for polymer replication by a combined method of femtosecond laser milling and CO_2_ laser polishing. They could achieve a surface roughness of less than 10 nm after the laser polishing process. However, the limitation of this method is that it is difficult to predict the exact profile of the output surface, since the subsequent laser polishing process will change the topography of the surface created by femtosecond laser milling in an uncertain way. Thus, to obtain a designed surface texture within a reasonable form error, extensive trial and error is needed. Other examples of combined machining methods that follow the paradigm of the rough-finish sequential process are laser ablation followed by electrical discharge machining (EDM) [21], femtosecond laser milling followed by FIB finishing [22], and electrochemical discharge machining (ECDM) followed by micro grinding [23]. To the authors’ knowledge, there is no research into the combined machining of laser ablation and diamond cutting, especially for machining brittle materials, such as single crystal silicon.

In order to improve the machining efficiency, as well as reduce diamond tool wear in fabricating lenticular lens silicon mold inserts, a new hybrid machining method combining laser ablation and diamond cutting was proposed in this research. This hybrid method also followed the paradigm of the rough-finish process. The laser ablation served as a rough machining process to remove the majority of the material, and the diamond cutting was the finishing process to shape the grooves, created by laser ablation, to their final status.

## 2. Materials and Methods

### 2.1. Experimental Process

**Figure 1:**
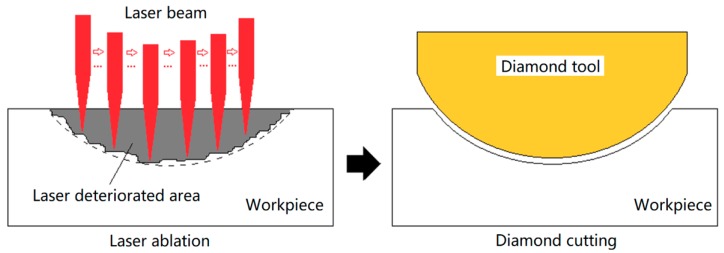
Illustration of the hybrid machining method.

Figure 1 illustrates the process of this hybrid machining method. First, the workpiece material was scanned by a high power nanosecond laser beam. By controlling the laser scanning parameters, grooves with pre-designed shapes were generated on the silicon workpiece surface. Second, the diamond tool was be aligned to the same position of the grooves generated by laser to remove the remaining material and perform finish cutting.

**Figure 2:**
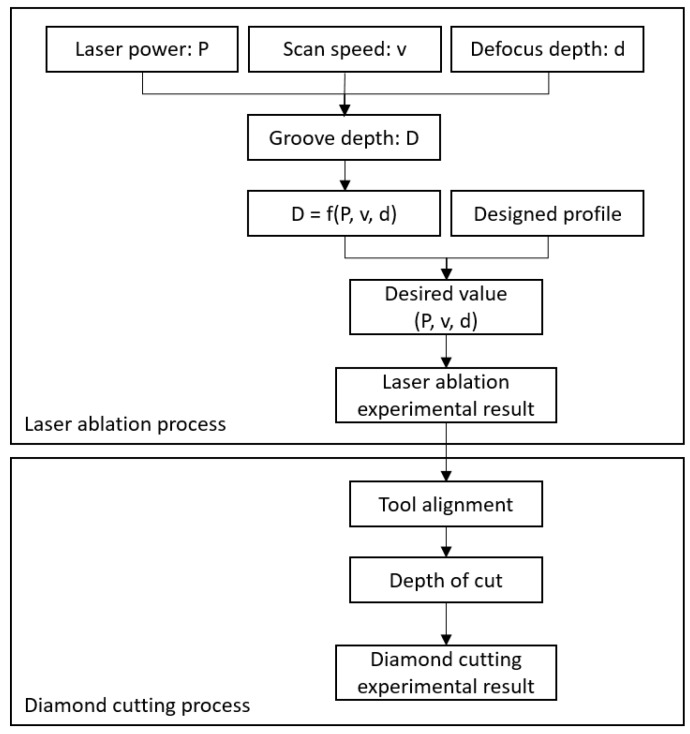
Flow chart of the hybrid machining process.

The flow chart of the hybrid machining process is shown in Figure 2. First, the influence of the laser ablation parameters, such as scan speed, laser power and defocus depth on ablation depth was investigated. Second, grooves with pre-designed shapes were generated by the laser ablation method with the optimized laser ablation parameters. Third, tool workpiece re-alignment and diamond cutting experiments were performed.

### 2.2. Laser Ablation Experiment

**Figure 3:**
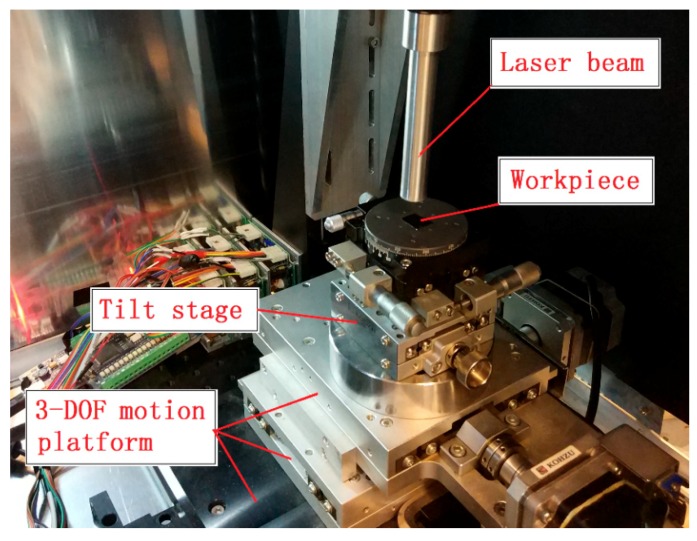
Laser micro-machining setup.

**Figure 4:**
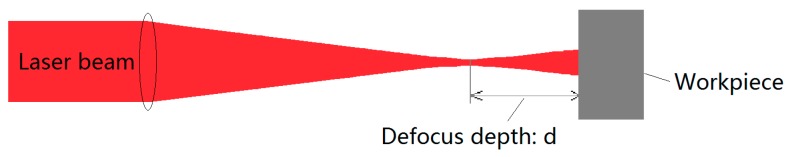
Illustration of the definition of defocus depth.

The laser ablation experiment was conducted on the homemade laser micro-machining setup developed by the authors, which is shown in Figure 3. The laser being used was a nanosecond pulsed laser with a wavelength of 2 µm. The pulse duration and repetition rate of this laser were 30 ns and 30 kHz, respectively. The rated average laser power was 10 W and the peak power was 10 kW. The workpiece material was single crystal silicon. In order to find the optimum laser machining parameters for a designed value of groove depth, the influence of laser scan speed, laser power, and defocus depth on ablation depth was investigated. The definition of the defocus depth is illustrated in Figure 4, and refers to the distance between the workpiece surface and laser beam focal point.

There are several methods to produce a groove of a given depth, such as changing laser power, scan speed or defocus depth. It is relatively easier to adjust the defocus depth by a numerically controlled 3-degree of freedom (DOF) motion platform than it is to adjust the laser power and scan speed. Therefore, in this experiment, different depths of laser ablation grooves was realized by changing the defocus depth.

**Figure 5:**
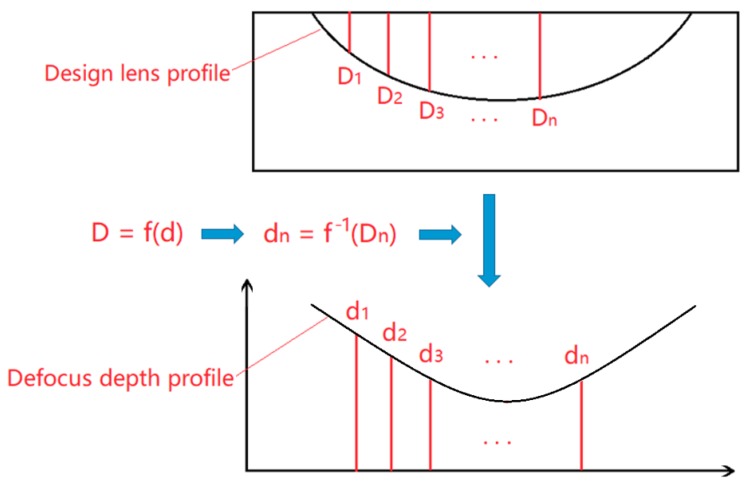
The procedure to determine defocus depth profile.

Figure 5 shows the procedure for determining the defocus depth profile. First, the actual depth of the designed lenticular lens at different positions, *D_n_*, was calculated. Then according to the relationship between the laser ablation depth and defocus depth obtained from the experimental result, the defocus depth, *d_n_*, was calculated. The interval between two laser scanning paths was set to be 5 µm.

### 2.3. Diamond Cutting Experiment

After the laser ablation process, grooves with a rough shape were obtained on a silicon workpiece surface. However, the surface quality of the laser-generated grooves was very unsatisfactory. In laser ablation, instead of directly removing it from the workpiece surface, the melt-off material piled up on the workpiece surface and subsequently needed to be removed by the diamond cutting process. Since the laser ablation experiment was performed on the laser micro-machining equipment, when the workpiece was moved to the diamond cutting machine, tool alignment was needed to make the groove axis comply with the diamond tool travel axis. Subsequently, diamond cutting with different finish depths of cuts was conducted to investigate the influence of the depth of cut on the surface quality of the machined grooves.

**Figure 6:**
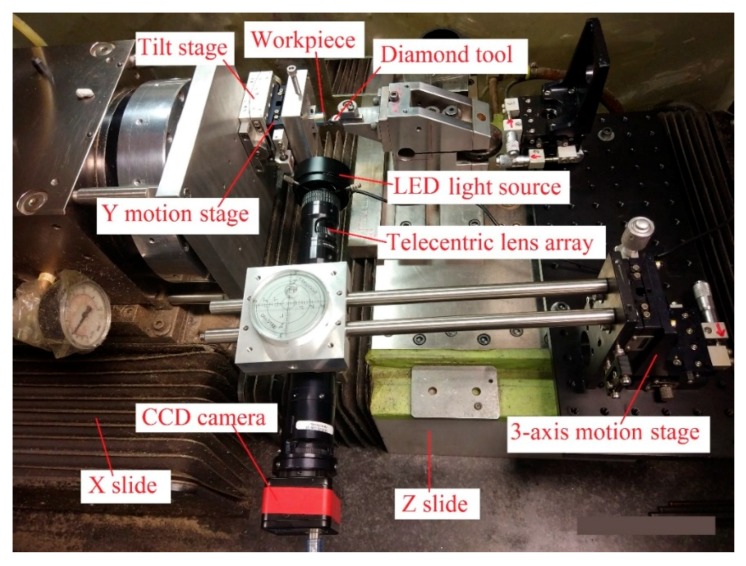
Diamond cutting experimental setup.

The diamond cutting experiment was performed on a Nanoform 200 ultra-precision lathe. Figure 6 shows the setup of the diamond cutting experiment. A charge-coupled device (CCD) camera was mounted on the lathe to assist with the alignment work. The tilt stage was used to tilt the workpiece surface to make sure the cutting process could be performed under a constant depth of cut. The Y motion stage was used to adjust the position of the workpiece along the vertical direction.

## 3. Results and Discussion

### 3.1. Laser Ablation Experimental Results

**Figure 7:**
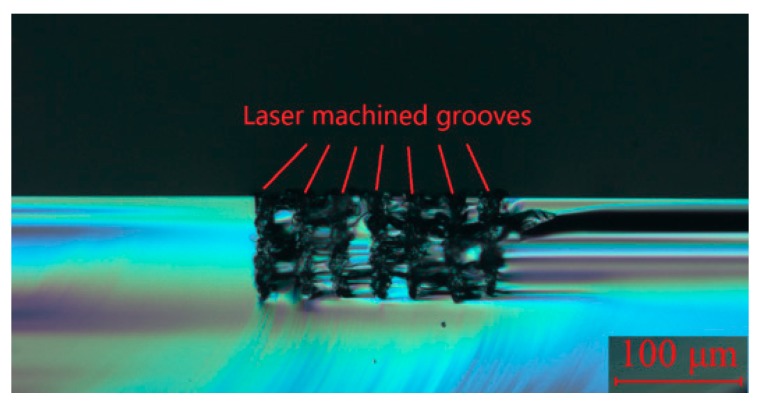
Cross-section view of laser-machined grooves on the silicon workpiece surface.

Figure 7 shows a typical optical microscopic image of the cross-sectional view of laser ablated grooves on a silicon workpiece surface. The laser power was 4.3 W and the scan speed was 50 mm/min in this case. The workpiece surface was adjusted to be at the focal point of the laser beam. All the grooves in this figure were generated with the same laser parameters.

**Figure 8:**
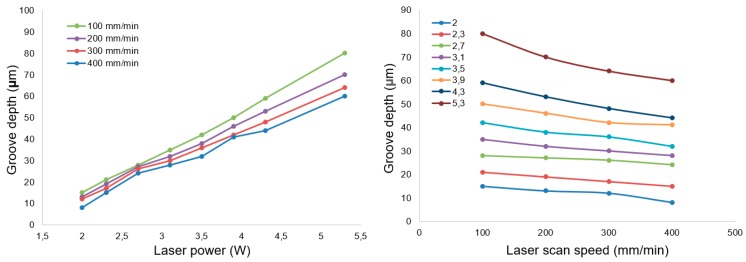
The influence of the laser power and scan speed on laser ablation depth.

It can be seen from this image that the depths of these laser-generated grooves were almost the same, which means the repeatability of the laser ablation process was reliable, and thus acceptable for our purpose. Laser ablation experiments with changing laser power and scan speed were conducted. Figure 8 shows the experimental results that indicated the influence of laser power and scan speed on laser ablation depth. A linear relationship between laser ablation depth and laser power can be seen. A linear relationship can similarly be seen between the laser ablation depth and scan speed.

**Figure 9:**
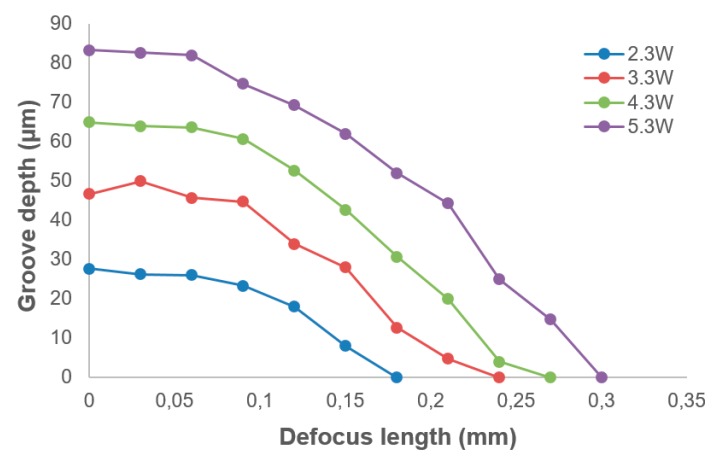
The influence of defocus depth on laser ablation depth.

Figure 9 shows the experimental results of the influence of defocus depth on the laser ablation depth. The laser scan speed was fixed to 400 mm/min for all experiments. Unlike the relationship between laser ablation depth and laser power, which could be depicted with a linear function, the influence of defocus depth on laser ablation depth was a non-linear effect.

**Figure 10:**
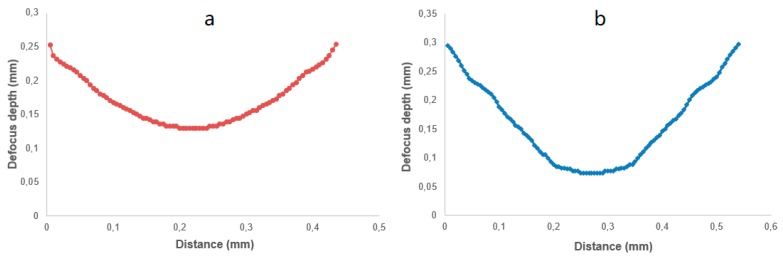
Defocus depth profiles for lenticular lens groove depths of (**a**) 50 µm and (**b**) 80 µm.

The cross-sectional profile of the lenticular lens groove was an arc. In this experiment, two grooves with different depths were generated in the laser ablation process. The design arc radius of the grooves was 0.5 mm, and the depths of the grooves were 50 µm and 80 µm. The width of the grooves were calculated to be 0.436 mm and 0.543 mm, respectively. The width of a single laser ablated groove was around 5 µm, which could be estimated from Figure 7. Thus, to generate one lenticular lens groove, tens or even more than one hundred repetitions of laser scanning passes were required. For the lenticular lens groove, the depths were different at different positions of the arc. In the experimental process, this was realized by changing the defocus depth. The procedure to determine the defocus depth profiles was shown in Figure 5. The defocus depth profiles for the designed lenticular lens groove depths of 50 µm and 80 µm were shown in Figure 10. The corresponding laser powers to produce these two grooves were 4.3 W and 5.3 W, respectively.

**Figure 11:**
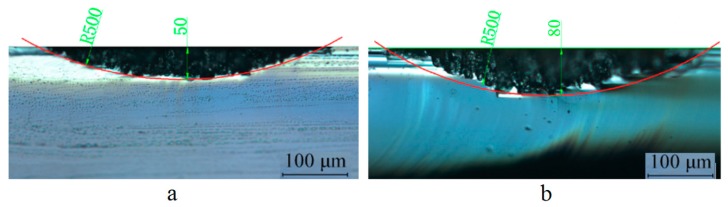
Cross-sectional profiles of laser-machined grooves with designed depth of (**a**) 50 µm and (**b**) 80 µm.

The optical microscopic images of the cross-sectional profiles of the laser-ablated grooves are shown in Figure 11. The red line shows the design lenticular lens profile. The experimental results agreed well with the design profiles.

### 3.2. Diamond Cutting Experimental Results

**Figure 12:**
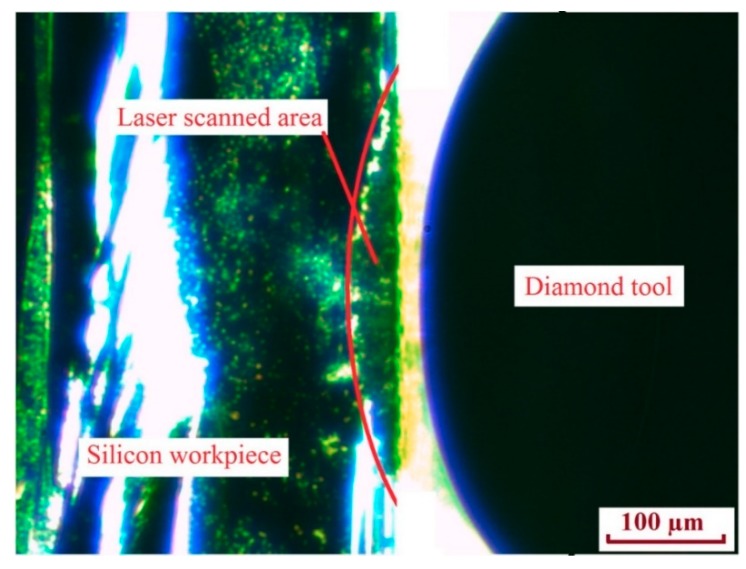
Charge-coupled device (CCD) camera captured image shows the tool alignment process.

Figure 12 shows an image of the tool alignment process captured by CCD camera. After the tool alignment work was completed, diamond cutting experiments were performed. The diamond cutting tool served as the shaper of grooves, previously generated by the laser, to give them the final shape. First, rough cutting was conducted to remove the laser deteriorated material that was still attached to the workpiece surface. The depth of the rough cut was set to be comparative to the laser grooved depth. Since the material in the region had been deteriorated by laser scanning and was no longer physically bonded to the base material, it could be removed easily without inducing large cracks on the machined workpiece surface, even when the depth of cut was as large as tens of micrometers. Second, finish cutting was performed to investigate the influence of the depth of cut on groove surface roughness.

**Figure 13:**
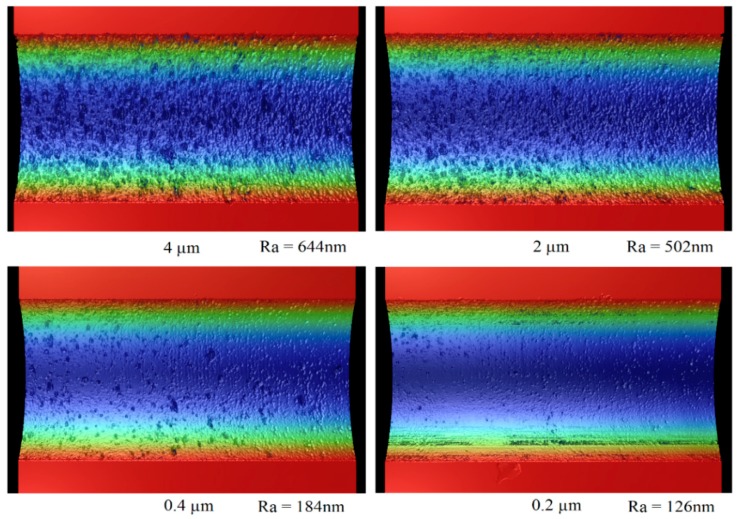
Groove surface profiles after finish cutting with different depths of cut.

Figure 13 shows the groove surface profiles measured by Zygo (Newview 8000, Zygo Corporation, Middlefield, CT, USA) after finish cutting with different depths of cuts. For all of the grooves, the total cutting depth was the same. For example, if the total finish cutting depth was 4 μm, it would need one pass for a depth of cut of 4 μm and twenty passes for a depth of cut of 0.2 μm.

**Figure 14:**
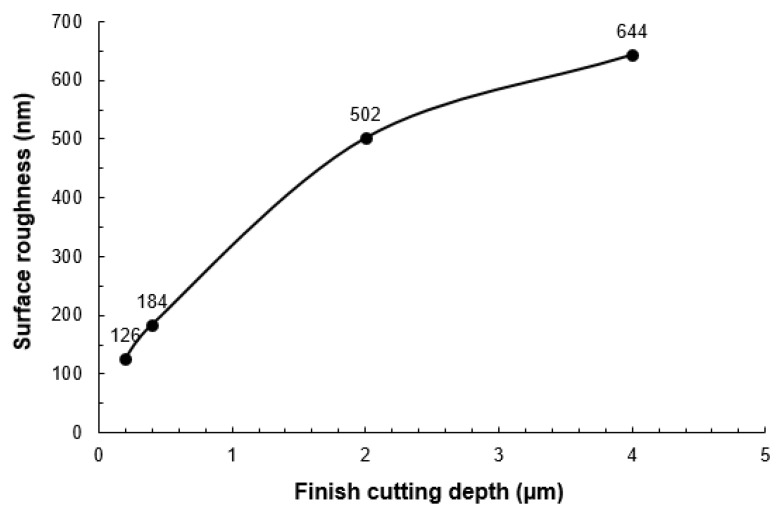
Relationship between depth of cut and surface roughness.

Figure 14 shows the relationship between the depth of cut and groove surface roughness. The surface roughness was measured on the bottom of the grooves with a sampling area of 250 μm × 250 μm. It can be seen that the surface roughness decreased with a decreasing depth of cut. When the depth of cut was 4 μm, large cracks frequently formed on the machined groove surface, which deteriorated the machined surface quality. When the depth of cut was 0.2 μm, the machined groove surface appeared to be much smoother with fewer and smaller cracks formed. The surface roughness was thus dramatically reduced compared with those machined with larger depths of cuts.

The main focus of this paper is on the feasibility of using this hybrid method to machine lenticular lens microstructures on a silicon workpiece surface. Since most of the material is removed in the laser ablation step, which is a relatively fast and low cost process, this machining method is expected to be highly efficient, as well as cost effective, compared to the conventional diamond cutting method. In this research, a preliminary experimental approach was successfully performed. The surface quality of the machined grooves was evaluated to demonstrate the feasibility of using this hybrid method to machine lenticular lens silicon mold inserts in terms of improving machining efficiency, as well as reducing tool wear.

However, there are limitations of this research. For example, even though the machined surface quality could be improved significantly by reducing the finish depth of the cut, the best surface roughness of the machined groove, which was in the order of 100 nm, still could not fulfill the requirement of a practically useful high quality lenticular lens mold insert. There are several potential methods to further improve the machining performance of this hybrid method, such as using a negative rake angle diamond tool, or further reducing the finish depth of the cut to below the critical depth of cut of single crystal silicon. Another limitation of this method is that, at present, the two machining steps were conducted separately on two independent machine systems, which brings about the problem of re-alignment before the second machining step was conducted. In the future, this can be solved by combining the two steps into one machine system.

Machining highly brittle materials using conventional methods is a long-term challenge. This work was a pilot research study of using hybrid methods to machine highly brittle materials. Further work is still required to make this method more applicable for the fabrication of lenticular lenses.

## 4. Conclusions

In this research, a hybrid machining method that combined laser ablation and diamond cutting was proposed to machine a lenticular lens silicon mold. A feasibility study was performed to investigate the feasibility of using this method to fabricate a lenticular lens silicon mold. The influence of laser power, laser scan speed and defocus depth on the laser ablation depth was investigated. It was found that there was a linear relationship between laser power and laser ablation depth, and between laser scan speed and laser ablation depth, whereas, the influence of defocus depth on laser ablation depth was a non-linear effect. In the diamond cutting process, the machined surface quality was able to be improved significantly by reducing the finish depth of the cut. The experimental results indicate that this could be a feasible method of manufacturing lenticular lens silicon molds or other similar microstructures with improved machining efficiency, as well as reduced tool wear.
